# Previous caesarean delivery is associated with adverse obstetric and perinatal outcomes: a real-world cohort study of 26,843 singleton pregnancies

**DOI:** 10.3389/fmed.2026.1915443

**Published:** 2026-10-01

**Authors:** Yi Yuan, Fan Wu, Jinfang Wang, Chuanmei Qin, Haoyi Jia, Yan Hou, Huiling Zhang, Shuqin Ma, Weihong Zeng, Yi Lin

**Affiliations:** 1 Reproductive Medicine Center, Shanghai Sixth People's Hospital Affiliated to Shanghai Jiao Tong University School of Medicine, Shanghai, China; 2 Shanghai Key Laboratory of Embryo Original Diseases, the International Peace Maternity & Child Health Hospital, Shanghai Jiao Tong University School of Medicine, Shanghai, China; 3 Obstetrics Department, General Hospital of Ningxia Medical University, Ningxia, China; 4 Department of Obstetrics and Gynecology, Shanghai General Hospital, Shanghai Jiao Tong University School of Medicine, Shanghai, China

**Keywords:** cohort study, effect modification, obstetric and perinatal outcomes, preterm birth, previous caesarean delivery

## Abstract

**Objective:**

To examine the association between a history of caesarean delivery and subsequent obstetric and perinatal outcomes.

**Design:**

A real-world retrospective cohort study was conducted at a large tertiary maternity hospital in Shanghai, China.

**Patients:**

Among 118,375 deliveries from January 2014 to August 2021, a total of 26,843 singleton pregnancies ≥28 weeks' gestation were included for analysis, comprising 11,359 women with a previous caesarean section (CS) and 15,484 with a prior vaginal delivery.

**Main outcomes:**

Obstetric and perinatal outcomes including placenta previa, placenta abruption, abnormal placental morphology, emergency CS, elective CS, gestational diabetes mellitus (GDM), preeclampsia (PE), postpartum hemorrhage, preterm birth, low birth weight, congenital malformation, neonatal asphyxia, and congenital infection.

**Results:**

After adjusting for relevant confounders, multivariable modified Poisson regression analyses showed that women with a previous CS history were associated with placenta previa [adjusted risk ratio (aRR): 1.71, 95% confidence interval (CI): 1.39–2.10], GDM (aRR: 1.28, 95% CI: 1.20–1.37), PE (aRR: 1.88, 95% CI: 1.56–2.27), elective CS (aRR: 1.35, 95% CI: 1.30–1.40), emergency CS (aRR: 1.24, 95% CI: 1.14–1.35), postpartum hemorrhage (aRR: 1.94, 95% CI: 1.45–2.58), preterm birth (aRR: 1.50, 95% CI: 1.36–1.65) and low birth weight (aRR: 1.37, 95% CI: 1.22–1.55). In sensitivity analyses using propensity score matching and multiple imputation, the associations remained consistent.

**Conclusion:**

A previous CS history is associated with adverse obstetric and perinatal outcomes, particularly placenta previa, GDM, PE, postpartum hemorrhage, preterm birth, and low birth weight. The association may vary by maternal age and history of miscarriage.

## 1 Introduction

Caesarean section (CS) rates have risen substantially worldwide over recent decades (1). In 2015, WHO revisited its 1985 recommendation and stated that the 10%–15% range should not be used as a target, and that CS should be performed when medically necessary (2). The reasons for the increased CS rates are multifactorial, including medical as well as non-medical factors (3–5). A lowered clinical indicator for performing an intrapartum CS, a growing number of childbearing women with advanced age, and maternal request without medical indications, have been counted among the leading causes (6–8). Although CS is a crucial intervention in life-threatening situations for both the mother and the newborn, it remains important to evaluate its association with women's and offspring's health.

Previous researches have shown that previous CS is associated with postpartum hemorrhage, placenta previa, preeclampsia (PE) and preterm birth in subsequent pregnancies (9–11). Cervical or uterine trauma incurred during CS may further predispose women to complications such as spontaneous preterm labor (12).

Despite these observations, most existing studies have been conducted in Western populations, and data from large cohorts in Asian settings remain limited. Moreover, the modifying effects of maternal characteristics on this association remain incompletely understood, and previous studies have yielded inconsistent findings regarding potential effect modifiers.

To address these gaps, we conducted a large retrospective cohort study involving 26,843 singleton deliveries among 118,375 in-patients in Shanghai, China. The aim was to evaluate the association between previous CS and obstetric and perinatal outcomes. In particular, we examined whether these associations differed by maternal age and history of miscarriage. Findings from this study may provide evidence to inform clinical management and antenatal surveillance for women with a history of CS.

## 2 Materials and methods

### 2.1 Study population and data collection

This retrospective cohort study was conducted at the International Peace Maternal and Child Health Hospital (IPMCH) of Shanghai Jiao Tong University School of Medicine, a tertiary care hospital in Shanghai of China. Delivery records from January 2014 to August 2021 were reviewed, comprising 118,375 births. The database included demographic characteristics, pregnancy complications, and delivery outcomes. Women who received prenatal care at IPMCH hospital but gave birth elsewhere were excluded from the analysis. This study was approved by the Institutional Ethics Committee of IPMCH (Number: GKLW 2021–17) and all procedures were conducted in accordance with the Declaration of Helsinki.

### 2.2 Inclusion and exclusion criteria

Eligible participants were women with: (1) singleton pregnancies with gestational age ≥28 weeks, and (2) a history of either previous CS or vaginal delivery. All included women had a history of exactly one previous delivery (either caesarean or vaginal). Women with multiple previous deliveries were excluded to minimize the confounding effects of repeated uterine scars and to isolate the specific association of a single prior caesarean delivery on subsequent pregnancy outcomes. The cutoff of 28 weeks' gestation was chosen because this is the standard threshold for defining preterm birth in the Chinese clinical setting, and outcomes of interest (e.g., placenta previa, placental abruption, preeclampsia, preterm birth) are clinically relevant only in pregnancies that have reached viability. Exclusion criteria were: (1) missing key information such as pre-pregnancy body mass index (BMI), education, miscarriage history; and (2) logical errors in the records, such as negative birth weight. Finally, a total of 26,843 women met the inclusion criteria, including 11,359 with a previous CS and 15,484 with a prior vaginal delivery.

### 2.3 Covariates at baseline

Baseline covariates included demographic and clinical factors: maternal age (categorized as <25 years, 25–30 years, 30–35 years, ≥35 years) (13), address (categorized as local, non-local), marital status (married or unmarried), payment method (health insurance or self-pay), pre-pregnancy BMI (underweight: BMI < 18.5 kg/m^2^; normal weight: 18.5 ≤ BMI < 24 kg/m^2^; overweight: 24 ≤ BMI < 28 kg/m^2^; obesity: BMI ≥ 28 kg/m^2^) (14), education level (below bachelor's, bachelor's, above bachelor's), mode of conception (natural conception, assisted reproduction), history of miscarriage, viral hepatitis, hyperthyroidism, hypothyroidism, asthma and anemia. The following covariates were included in the multivariable models: maternal age, pre-pregnancy BMI, education level, miscarriage history, mode of conception, medical history of anemia, hypothyroidism, insurance status, ethnicity (Han vs. non-Han), marital status, and local residence. Covariates were selected *a priori* based on clinical plausibility. Maternal age and pre-pregnancy BMI were included because they are well-established risk factors for adverse pregnancy outcomes; education level and insurance status were included as proxies for socioeconomic status; miscarriage history and mode of conception were included due to their known associations with placental complications; and medical history of anemia and hypothyroidism were included because they may influence both the likelihood of caesarean delivery and subsequent pregnancy outcomes.

### 2.4 Outcome definition

The outcomes were categorized into obstetric complications and perinatal outcomes, including placenta previa, placental abruption, abnormal placental morphology, postpartum hemorrhage, PE, gestational diabetes mellitus (GDM), emergency CS, elective CS, preterm birth, low birth weight, sex of newborn infant, neonatal asphyxia, congenital malformation, and congenital infection. Placental abruption was defined as complete or partial separation of the placenta from the uterine wall before delivery (15). Abnormal placental morphology included circumvallate placenta, bilobed placenta, accessory placenta and velamentous cord insertion (16), these conditions were grouped as a composite outcome because they share a common pathophysiological mechanism of abnormal placentation. The frequency of each individual component is presented in Supplementary Table S1. Placenta previa was defined as the placenta attached to the lower uterine segment lower than the fetal presentation, with the placental margin reaching or covering the cervical os after 28 weeks of gestation (17). PE was defined as new-onset hypertension (systolic blood pressure ≥140 mmHg and/or diastolic blood pressure ≥90 mmHg) and proteinuria (≥300 mg/day) with or without abnormalities across multiple systems such as liver, kidney, nerve and blood system after 20 weeks of gestation (18). Postpartum hemorrhage was defined as blood loss ≥500 mL after vaginal birth or ≥1,000 mL after caesarean delivery (19). Preterm birth was defined as delivery from 28 to 36^+6^ weeks gestation (20). Gestational age was estimated based on the first day of the last menstrual period. Low birth weight was defined as birth weight ≤2,500 g (21). GDM was defined as any degree of glucose intolerance with onset or first recognition during pregnancy (22). Neonatal asphyxia was defined as the failure to establish breathing following delivery (23). Congenital malformation included aneuploidy. Congenital infection included TORCH infections (toxoplasmosis, rubella, cytomegalovirus, herpes simplex virus) and other congenital infections. All outcomes were identified from hospital discharge diagnoses coded using the ICD-10 system and recorded by attending physicians.

### 2.5 Statistical analyses

Baseline characteristics of the study population were compared between women with a previous caesarean delivery and those with a previous vaginal delivery using chi-square tests for categorical variables. Standardized mean differences (SMD) were calculated to quantify the magnitude of differences between the two groups, with SMD < 0.1 indicating good balance. To evaluate the independent association between previous caesarean delivery and each obstetric and perinatal outcome, we performed multivariable modified Poisson regression with robust error variance to estimate adjusted risk ratios (aRR) and 95% confidence intervals (CI). The multivariable models included the following covariates: maternal age, BMI, education level, miscarriage history, anemia, hypothyroidism, insurance status, marital status, local residence, mode of conception, and ethnicity.

To further address potential confounding and assess the robustness of our findings, we performed a sensitivity analysis using propensity score matching (PSM). Propensity scores were estimated using a logistic regression model that included all covariates listed above. We then performed 1 : 1 nearest-neighbor matching with a caliper of 0.2. Balance of covariates after matching was assessed using SMD, with values <0.1 indicating good balance (Table 1). In the matched cohort, we estimated unadjusted risk ratios (RR) and 95% CI using modified Poisson regression with only the grouping variable (previous caesarean delivery vs. previous vaginal delivery) as the independent variable (Table 2).

**Table 1:** Baseline sociodemographic characteristics and medical history of pregnant women before and after propensity score matching.

Characteristic	Previous vaginal delivery (*n* = 15,484)	Before PSM		SMD	Previous vaginal delivery (*n* = 11,309)	After PSM		SMD
		Previous caesarean delivery (*n* = 11,359)	*P* value			Previous caesarean delivery (*n* = 11,309)	*P* value	
Insurance	11,930 (77.05)	8,378 (73.76)	<0.001	0.076	8,449 (74.71)	8,374 (74.05)	0.26	0.015
Ethnicity-Han	15,273 (98.64)	11,159 (98.24)	<0.01	0.032	11,125 (98.37)	11,110 (98.24)	0.471	0.01
Single marital statusa	72 (0.46)	102 (0.90)	<0.001	0.053	70 (0.62)	95 (0.84)	0.061	0.026
Local	10,762 (69.50)	7,642 (67.28)	<0.001	0.048	7,649 (67.63)	7,638 (67.54)	0.887	0.002
Maternal age								
<25	111 (0.71)	59 (0.52)	<0.001	0.205	50 (0.44)	59 (0.52)	0.792	0.014
25–30	2,281 (14.73)	1,085 (9.55)			1,087 (9.61)	1,085 (9.59)		
30–35	7,402 (47.80)	5,092 (44.83)			5,055 (44.70)	5,090 (45.01)		
≥35	5,690 (36.75)	5,123 (45.10)			5,117 (45.25)	5,075 (44.88)		
BMI			<0.001	0.178				
Underweight <18.5	1,741 (11.24)	972 (8.56)			976 (8.63)	972 (8.59)	<0.001	0.073
Normal: 18.5–24	11,556 (74.63)	8,112 (71.41)			8,367 (73.99)	8,083 (71.47)		
Overweight: 24–28	1,861 (12.02)	1,832 (16.13)			1,650 (14.59)	1,828 (16.16)		
Obesity: ≥28	326 (2.11)	443 (3.90)			316 (2.79)	426 (3.77)		
Education			<0.001	0.125				
Below university	4,494 (29.02)	3,734 (32.87)			3,726 (32.94)	3,690 (32.63)	0.371	0.019
University	7,544 (48.72)	5,620 (49.48)			5,518 (48.79)	5,616 (49.66)		
Graduate school	3,446 (22.26)	2,005 (17.65)			2,065 (18.26)	2,003 (17.71)		
Assisted reproductive technologies	270 (1.74)	235 (2.07)	0.015	0.036	236 (2.09)	233 (2.06)	0.693	0.011
Miscarriage history	7,950 (51.34)	6,602 (58.1)	<0.001	0.136	6,541 (57.84)	6,553 (57.94)	0.882	0.002
Viral hepatitis	505 (3.26)	340 (2.99)	0.227	0.015	359 (3.17)	340 (3.01)	0.489	0.01
Hyperthyroidism	112 (0.72)	74 (0.65)	0.531	0.009	95 (0.84)	74 (0.65)	0.123	0.022
Hypothyroidism	164 (1.06)	52 (0.46)	<0.001	0.069	43 (0.38)	52 (0.45)	0.411	0.012
Asthma	26 (0.17)	27 (0.24)	0.257	0.016	19 (0.17)	27 (0.24)	0.302	0.016
Anemia	2,252 (14.54)	1,917 (16.88)	<0.001	0.064	1,876 (16.59)	1,914 (16.92)	0.51	0.009

**Table 2:** Obstetric and perinatal outcomes in women with a previous caesarean section compared with a previous vaginal delivery.

	Before PSM			After PSM		
	Previous vaginal delivery^c^	Previous caesarean delivery^c^	Multivariable regression models aRR [95% CI]^a^	Previous vaginal delivery^c^	Previous caesarean delivery^c^	Multivariable regression models^b^ RR [95% CI]^b^
Obstetric outcomes						
Placenta previa	149 (0.96)	227 (1.99)	1.71 (1.39–2.10)	126 (1.11)	224 (1.98)	1.78 (1.43–2.21)
Placental abruption	294 (1.89)	230 (2.02)	0.95 (0.80–1.13)	229 (2.02)	229 (2.02)	1.00 (0.83–1.20)
Abnormal placental morphology	98 (0.63)	47 (0.41)	0.60 (0.42–0.85)	85 (0.75)	47 (0.41)	0.55 (0.39–0.79)
GDM	1,795 (11.59)	1,838 (16.18)	1.28 (1.20–1.37)	1,596 (14.11)	1,829 (16.17)	1.15 (1.07–1.23)
Preeclampsia (PE)	177 (1.14)	291 (2.56)	1.88 (1.56–2.27)	166 (1.46)	289 (2.55)	1.74 (1.44–2.11)
Perinatal outcomes						
Postpartum hemorrhage	78 (0.50)	123 (1.08)	1.94 (1.45–2.58)	57 (0.50)	121 (1.07)	2.12 (1.55–2.91)
Preterm birth <37 week	753 (4.86)	897 (7.89)	1.50 (1.36–1.65)	555 (4.91)	889 (7.86)	1.60 (1.44–1.78)
Emergency CS	1,163 (7.51)	1,114 (9.80)	1.24 (1.14–1.35)	893 (7.90)	1,109 (9.81)	1.24 (1.14–1.36)
Elective CS	5,712 (36.88)	5,752 (50.64)	1.35 (1.30–1.40)	4,197 (37.11)	5,730 (50.67)	1.37 (1.31–1.42)
Sex, boy	8,101 (52.31)	5,832 (51.34)	0.98 (0.95–1.02)	5,893 (52.10)	5,807 (51.35)	0.99 (0.95–1.02)
Low birth weight (<2,500 g)	496 (3.20)	545 (4.79)	1.37 (1.22–1.55)	370 (3.27)	540 (4.77)	1.46 (1.28–1.67)
Congenital malformation	71 (0.46)	44 (0.39)	0.82 (0.56–1.20)	56 (0.49)	44 (0.39)	0.79 (0.53–1.17)
Neonatal asphyxia	224 (1.44)	146 (1.28)	0.83 (0.67–1.02)	157 (1.39)	146 (1.29)	0.93 (0.74–1.17)
Congenital infection	150 (0.96)	81 (0.71)	0.69 (0.52–0.91)	125 (1.11)	80(0.71)	0.64 (0.48–0.85)

GDM, gestational diabetes mellitus. aRR, adjusted risk ratio; RR, risk ratio; CI, confidence interval; CS, caesarean section; PSM, propensity score matching.

a For the before PSM analysis (left panel), aRRs were estimated using multivariable modified Poisson regression with robust error variance, adjusted for maternal age (categorized as <25, 25–30, 30–35, ≥35 years), pre-pregnancy BMI, education level, miscarriage history, mode of conception, medical history of anemia and hypothyroidism, insurance status, ethnicity, marital status, and local residence.

b For the after PSM analysis (right panel), RRs were estimated using unadjusted modified Poisson regression in the matched cohort, as all covariates were well-balanced after matching (SMD < 0.1 for all variables). The matched cohort comprised 11,309 pairs.

c Data are presented as *n* (%) for incidence columns.

In addition to PSM, we addressed missing data as a separate sensitivity analysis. Missing data were present for BMI, education, and miscarriage history. Assuming data were missing at random, we performed multiple imputation using chained equations (mice package in R, m = 5), with predictive mean matching for continuous variables and logistic regression for categorical variables. The imputation model included all variables in the final analysis. Estimates from the imputed datasets were pooled using Rubin's rules and compared with the complete-case analysis (Supplementary Table S2).

To assess whether the association between previous caesarean delivery and adverse outcomes differed across subgroups, we tested multiplicative interactions by maternal age (<35 vs. ≥35 years) and history of miscarriage (no vs. yes) using modified Poisson regression with robust error variance. Interaction *P* values were calculated for all outcomes. For outcomes with a significant interaction (*P* < 0.05), we performed stratified analyses using multivariable Poisson regression with robust error variance within each stratum, adjusting for the same covariates as the primary analysis except for the stratifying variable itself. Results were visualized using forest plots.

All statistical analyses were performed using R version 4.3.0. A two-sided *P* value < 0.05 was considered statistically significant.

## 3 Results

### 3.1 Baseline characteristics and distribution of covariates

A total of 26,843 singleton pregnant women with gestation ≥28 weeks were included in data analysis, among whom 11,359 cases had a previous CS history and 15,484 controls had a history of vaginal delivery (Figure 1). Baseline characteristics of the participants were summarized in Table 1. Compared with the vaginal delivery group, women with a prior CS were older, single marital (0.90% vs. 0.46%; *P* < 0.001), and overweight and obese (20.03% vs. 14.13%; *P* < 0.001), as well as have a lower educational level (below university: 32.87% vs. 29.02%; *P* < 0.001), a history of miscarriage (58.1% vs. 51.34%; *P* < 0.001) and received assisted reproductive technologies (2.07 vs. 1.74, *P* = 0.015). Those with a CS history were less likely to have health insurance (73.76% vs. 77.05%; *P* < 0.001), Han ethnicity (98.24% vs. 98.64%, *P* *<* 0.01) and hypothyroidism (0.46% vs. 1.06%, *P* < 0.001), and reside locally (67.28% vs. 69.50%, *P* < 0.001).

**Figure 1:**
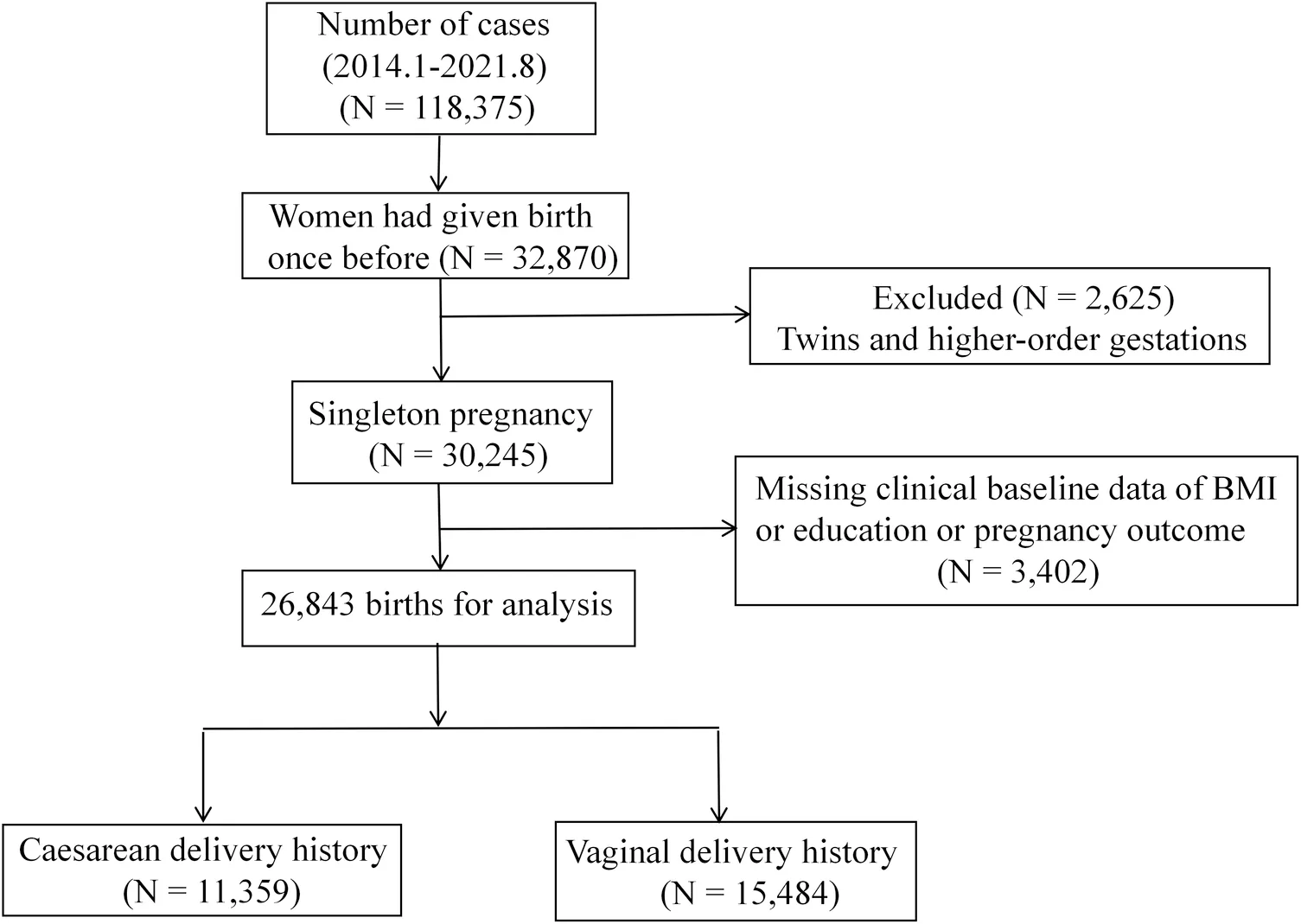
A flowchart of sample selection process. This retrospective cohort study utilized 118,375 deliveries between January 2014 and August 2021. After inclusion and exclusion process, a total of 26,843 singleton pregnant women were included in data analysis, among whom 11,359 cases had a CS history and 15,484 controls had a history of vaginal delivery.

### 3.2 Obstetric and perinatal outcomes

The descriptive analyses and univariable or multivariable modified Poisson regression analyses of obstetric and perinatal outcomes are summarized in Table 2. The descriptive analyses showed that women with a history of CS had higher incidences of placenta previa (1.99% vs. 0.96%), GDM (16.18% vs. 11.59%), PE (2.56% vs. 1.14%), postpartum hemorrhage (1.08% vs. 0.50%), preterm birth (7.89% vs. 4.86%), emergency CS (9.80% vs. 7.51%), elective CS (50.64% vs. 36.88%) and low birth weight (4.79% vs. 3.20%) in current pregnancies. Consistently, multivariable analyses indicated that previous caesarean delivery was associated with placenta previa (aRR: 1.71, 95% CI: 1.39–2.10), GDM (aRR: 1.28, 95% CI: 1.20–1.37), PE (aRR: 1.88, 95% CI: 1.56–2.27), postpartum hemorrhage (aRR: 1.94, 95% CI: 1.45–2.58), preterm birth (aRR: 1.50, 95% CI: 1.36–1.65), low birth weight (aRR:1.37, 95% CI: 1.22–1.55), elective CS (aRR: 1.35, 95% CI: 1.30–1.40) and emergency CS (aRR: 1.24, 95% CI: 1.14–1.35) in subsequent pregnancies. The high aRR for elective CS is expected, as women with previous CS are more likely to undergo repeat cesarean delivery.

To assess the robustness of our findings, we performed propensity score matching as a sensitivity analysis. The results from the matched cohort were consistent with those from the primary complete-case analysis (Table 2), supporting the stability of our conclusions. The matched cohort comprised 11,309 pairs with well-balanced baseline characteristics (Table 1).

We further assessed the potential influence of missing data. Missing data proportions were 10.16% vs. 10.82% for BMI, 8.70% vs. 9.61% for education, and 0.07% vs. 0.05% for miscarriage history in the previous vaginal delivery and previous CS groups, respectively. Multiple imputation sensitivity analysis yielded estimates consistent with the primary complete-case analysis for all outcomes (Supplementary Table S2), supporting the robustness of our findings.

To further clarify the association between previous CS and postpartum hemorrhage, we stratified the analysis by current delivery mode. Among women who delivered vaginally, previous CS was associated with an aRR of 2.54 (95% CI: 1.69–3.81) for PPH; among those who delivered by caesarean section, the corresponding aRR was 1.59 (95% CI: 1.06–2.40). The association remained significant in both strata (Table 3).

**Table 3:** Stratified analysis of postpartum hemorrhage by current delivery mode.

Current delivery mode	Previous vaginal delivery	Previous cesarean delivery	aRR (95% CI)
Vaginal delivery	41/8,609 (0.48)	57/4,493 (1.27)	2.54 (1.69–3.81)
Caesarean delivery	37/6,875 (0.54)	66/6,866 (0.96)	1.59 (1.06–2.40)

Data are presented as *n* (%). aRR, adjusted risk ratio; CI, confidence interval; CS, caesarean section; PPH, postpartum hemorrhage.

aRRs were estimated using multivariable modified Poisson regression with robust error variance, adjusted for maternal age, pre-pregnancy BMI, education level, miscarriage history, anemia, hypothyroidism, insurance status, marital status, local residence, mode of conception, and ethnicity. The analysis was stratified by current delivery mode.

### 3.3 Stratified analyses

We assessed effect modification by maternal age and history of miscarriage using interaction tests. Interaction *P* values for all outcomes are presented in Supplementary Table S3. For outcomes with a significant interaction, stratified analyses were performed.

Among women aged <35 years, previous CS was associated with GDM (aRR = 1.39, 95% CI: 1.26–1.53) and preterm birth (aRR = 1.32, 95% CI: 1.14–1.53), but not with emergency CS (aRR = 1.09, 95% CI: 0.97–1.23) or neonatal asphyxia (aRR = 1.12, 95% CI: 0.85–1.48). Among women aged ≥35 years, previous CS was associated with GDM (aRR = 1.22, 95% CI: 1.11–1.33), preterm birth (aRR = 1.73, 95% CI: 1.51–1.97) and emergency CS (aRR = 1.46, 95% CI: 1.30–1.65), and was inversely associated with neonatal asphyxia (aRR = 0.55, 95% CI: 0.40–0.77) (Figure 2, Supplementary Table S4).

**Figure 2:**
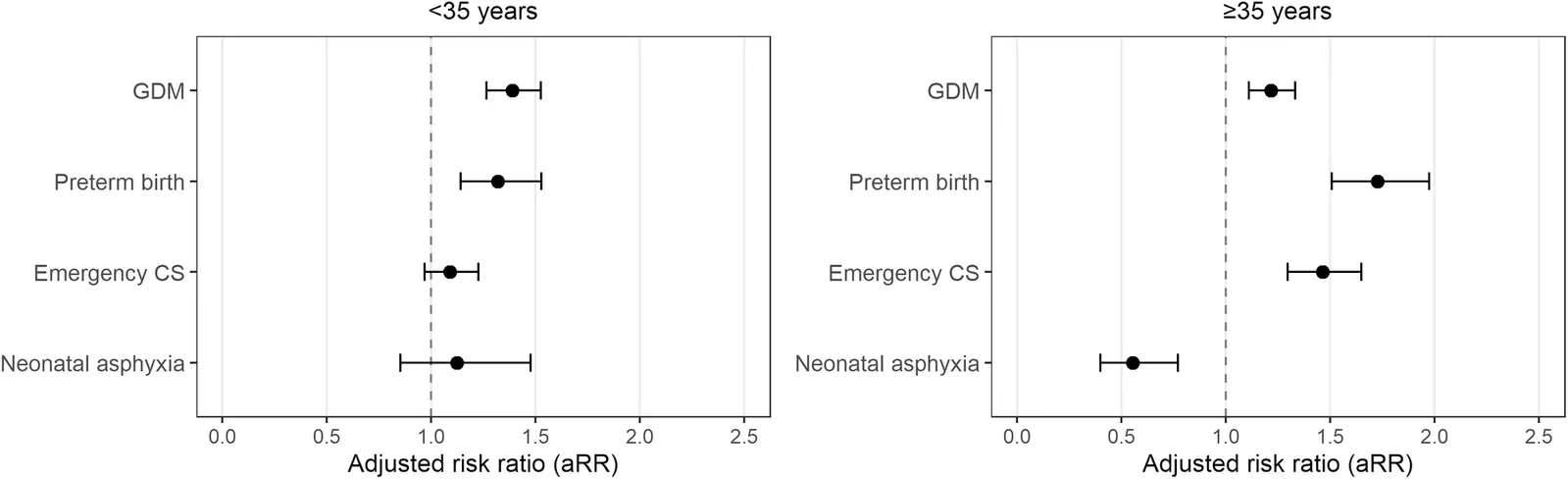
Stratified analysis of the association between previous caesarean delivery and adverse outcomes by maternal age. Adjusted risk ratios (aRR) with 95% confidence intervals are shown for women aged <35 years (left panel) and ≥35 years (right panel). All models were adjusted for BMI, education, anemia, hypothyroidism, insurance, marital status, residence, mode of conception, miscarriage history and ethnicity except for the stratifying variable itself. GDM, gestational diabetes mellitus; CS, caesarean section.

Among women without a history of miscarriage, previous CS was associated with preterm birth (aRR = 1.26, 95% CI: 1.07–1.49), and showed an inverse association with placental abruption (aRR = 0.70, 95% CI: 0.49–0.98), but was not associated with preeclampsia, emergency CS, or low birth weight. Among women with a history of miscarriage, previous CS was associated with preeclampsia (aRR = 2.41, 95% CI: 1.91–3.04), preterm birth (aRR = 1.76, 95% CI: 1.56–2.00), emergency CS (aRR = 1.49, 95% CI: 1.34–1.66), and low birth weight (aRR = 1.70, 95% CI: 1.46–1.99) (Figure 3, Supplementary Table S5).

**Figure 3:**
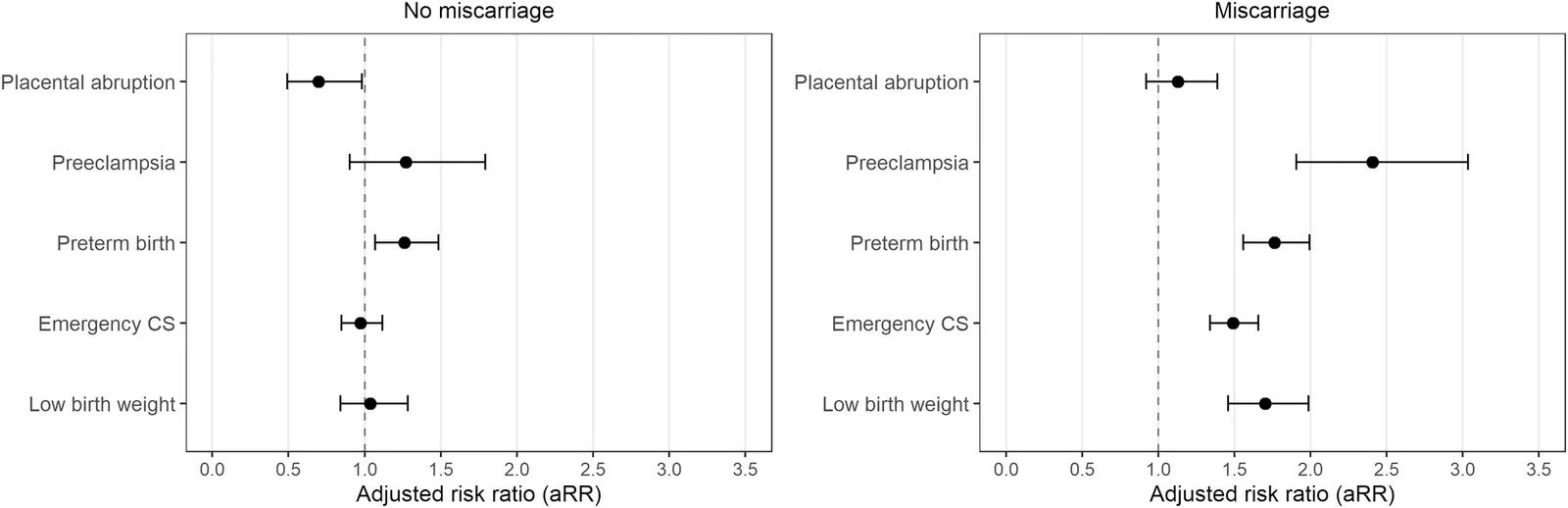
Stratified analysis of the association between previous caesarean delivery and adverse outcomes by history of miscarriage. Adjusted risk ratios (aRR) with 95% confidence intervals are shown for women without a history of miscarriage (left panel) and with a history of miscarriage (right panel). All models were adjusted for BMI, education, anemia, hypothyroidism, insurance, marital status, residence, mode of conception, maternal age and ethnicity except for the stratifying variable itself. CS, caesarean section.

## 4 Discussion

The aim of this study was to investigate the association of a prior CS on subsequent obstetric and neonatal outcomes beyond 28 weeks of gestation. We defined the CS rate as the proportion of caesarean deliveries relative to the total number of live births in a given period (24). In our cohort, there were 13,741 caesarean deliveries out of a total of 26,843 births, yielding a CS rate of 51.19%. Globally, the CS rate was approximately 21.1% between 2010 and 2018 and continued to rise annually, and projections indicate that by 2030, an estimated 28.5% of women worldwide will deliver via CS (25). The 2015 WHO statement clarified that CS rates should be determined by clinical need rather than a fixed threshold (2). The high CS rate may be partly explained by the implementation of China's two-child policy, an increase in maternal age, and sociocultural factors such as maternal preference for selecting delivery dates based on superstition or religious beliefs. Therefore, it is imperative to investigate the implications of previous caesarean delivery on both maternal and neonatal health outcomes in the subsequent pregnancies.

A retrospective cohort study has indicated that a prior CS is associated with placenta previa (RR: 1.47; 95% CI: 1.41–1.52) (26), which is consistent with our findings. In alignment with our results, previous studies demonstrate that women with a prior CS are more likely to experience adverse perinatal outcomes in subsequent pregnancies, such as preterm delivery (10, 27). We speculate that cervical trauma incurred during caesarean delivery may compromise cervical integrity, thereby predisposing to spontaneous preterm birth. However, this threshold excludes pregnancies that ended before 28 weeks, including those with severe placental complications leading to early pregnancy loss. Consequently, our findings may not be generalizable to pregnancies with extremely preterm outcomes, and the association between prior CS and placental disorders may be underestimated if such early losses were more common in women with previous CS. Furthermore, previous caesarean delivery has been reported to be associated with a two- to seven-fold increase in postpartum PE compared to vaginal birth (28), the underlying pathophysiological mechanisms remain unclear, but it is postulated that defective endometrial healing following CS may impair trophoblast invasion, reduce uteroplacental vascularization, and result in incomplete spiral artery remodeling. For women with a history of caesarean delivery, placental function and blood pressure should be monitored more closely. Women with a history of CS were much more likely to undergo repeat caesarean in our cohort, which largely reflects clinical management decisions rather than biological complications.

The significant interactions observed in our study suggest that the association between previous CS and certain adverse outcomes may vary by maternal age and history of miscarriage. Women with a history of miscarriage appeared to be particularly susceptible to the adverse associations of previous CS with placental and perinatal outcomes, with significantly stronger associations with preeclampsia, preterm birth, emergency CS, and low birth weight compared with those without such history. This may reflect underlying endometrial or decidual abnormalities that predispose to both recurrent miscarriage and subsequent placental dysfunction. In contrast, the inverse association between previous CS and neonatal asphyxia in women aged ≥35 years, as well as the inverse association with placental abruption in women without miscarriage history, should be interpreted with caution given the relatively small number of events and the possibility of residual confounding. These findings should be considered exploratory and warrant further investigation in larger studies.

The principal strength of our study lies in its large sample size (11,359 cases with a previous CS history vs. 15,484 controls with a previous vaginal delivery), which enhances the statistical power and generalizability of the findings. Moreover, we adjusted for a range of potential confounders.

Nevertheless, several limitations must be acknowledged. First, the retrospective design precludes causal inference, and the use of electronic medical records may have introduced misclassification bias, although outcomes were identified from ICD-10 coded discharge records. Second, due to limitations in medical record documentation, we were unable to distinguish between prior elective and emergency caesarean deliveries, which may have differential associations with subsequent obstetric and perinatal outcomes. Third, our study did not assess long-term outcomes among offspring, which should be addressed in future studies. Fourth, although we adjusted for a range of confounders, residual confounding cannot be fully excluded, as our database lacked information on indication for primary CS, incision type, interpregnancy interval, and other unmeasured factors that may have contributed to the observed associations. Fifth, although missing data were addressed using multiple imputation, residual bias due to missingness cannot be fully excluded. Finally, our findings are based on a single-center cohort with a predominantly Han Chinese population, which may limit generalizability to other settings. Multicenter and prospective studies are needed to validate these findings.

## 5 Conclusion

In conclusion, our data suggest that a history of caesarean section is associated with adverse obstetric and perinatal outcomes in subsequent pregnancies, including placenta previa, GDM, PE, postpartum hemorrhage, preterm birth and low birth weight. These associations were robust in the main analysis and remained consistent in sensitivity analyses. However, associations with neonatal outcomes (e.g., congenital malformation, congenital infection) were not statistically significant, indicating that prior CS may have a more limited association with neonatal health. Given the observed effect modification, the influence of maternal age and history of miscarriage warrants further investigation.

## Data Availability

The datasets presented in this article are not readily available because The datasets generated and analysed during the current study are not publicly available due to restrictions stipulated by the hospital ethics committee and to protect patient privacy, but are available from the corresponding author on reasonable request and with permission from the International Peace Maternal and Child Health Hospital (IPMCH). Requests to access the datasets should be directed to Yi Lin, yilinonline@126.com.
