# Supplementary material for: Previous caesarean delivery is associated with adverse obstetric and perinatal outcomes: a real-world cohort study of 26,843 singleton pregnancies
Source: Front Med (Lausanne). 2026 Oct 1;13:1915443. doi: 10.3389/fmed.2026.1915443 (PMC13668819; doi:10.3389/fmed.2026.1915443)
Supplement: Supplementary file 1 [file Table1.docx]

**Supplementary Table S1. Frequency of individual components of abnormal placental morphology.**

| Component |  | Previous vaginal delivery (n = 15,484) | Previous caesarean delivery (n = 11,359) |
| --- | --- | --- | --- |
| Circumvallate placenta |  | 2(0.01) | 0(0.00) |
| Bilobed placenta |  | 2(0.01) | 2(0.02) |
| Accessory placenta |  | 21(0.14) | 14(0.12) |
| Velamentous cord insertion |  | 77(0.50) | 33(0.29) |
| Total |  | 98(0.63) | 47(0.41) |

Data are presented as n (%). Components are not mutually exclusive; a single pregnancy may have more than one component. Total represents the number of women with at least one component, and may be smaller than the sum of individual components.

**Supplementary Table S2. Sensitivity analysis using multiple imputation (m = 5).**

| Outcome | All population  aRR (95% CI) | Sensitivity analysis  aRR (95% CI) |
| --- | --- | --- |
| Obstetric outcomes |  |  |
| Placenta previa | 1.71 (1.39-2.10) | 1.82 (1.50–2.20) |
| Placental abruption | 0.95 (0.80-1.13) | Not converged |
| Abnormal placental morphology | 0.60 (0.42-0.85) | Not converged |
| GDM | 1.28 (1.20-1.37) | 1.29 (1.21–1.37) |
| Preeclampsia (PE) | 1.88 (1.56-2.27) | 1.94 (1.63–2.31) |
| Perinatal outcomes |  |  |
| Postpartum hemorrhage | 1.94 (1.45-2.58) | 2.14 (1.65–2.77) |
| Preterm birth < 37 week | 1.50 (1.36-1.65) | 1.52 (1.38–1.66) |
| Emergency CS | 1.24 (1.14-1.35) | 1.30 (1.20–1.41) |
| Elective CS | 1.35 (1.30-1.40) | 1.40 (1.35–1.45) |
| Sex, boy | 0.98 (0.95-1.02) | 0.98 (0.95–1.02) |
| Low birth weight (< 2500 g) | 1.37 (1.22-1.55) | 1.42 (1.27–1.60) |
| Congenital malformationd | 0.82 (0.56-1.20) | 0.89 (0.64–1.22) |
| Neonatal asphyxia | 0.83 (0.67-1.02) | 0.87 (0.71–1.05) |
| Congenital infectione | 0.69 (0.52-0.91) | 0.60 (0.47–0.76) |

aRR, adjusted risk ratio; CI, confidence interval; CS, caesarean section; GDM, gestational diabetes mellitus.

All population: complete-case multivariable modified Poisson regression.

Sensitivity analysis: multiple imputation with chained equations (m = 5), assuming missing at random. Placental abruption and abnormal placental morphology models failed to converge due to the small number of events and were not included.

**Supplementary Table S3. Interaction P values for maternal age and history of miscarriage across all outcomes.**

| Outcome |  | *P* for interaction | | |
| --- | --- | --- | --- | --- |
|  |  | Age |  | Miscarriage |
| Placenta previa |  | 0.301 |  | 0.549 |
| Placental abruption |  | 0.234 |  | 0.020 |
| Abnormal placental morphology |  | 0.295 |  | 0.129 |
| GDM |  | 0.003 |  | 0.864 |
| Preeclampsia (PE) |  | 0.739 |  | 0.001 |
| Postpartum hemorrhage |  | 0.341 |  | 0.319 |
| Preterm birth < 37 week |  | 0.036 |  | <0.001 |
| Emergency CS |  | 0.003 |  | <0.001 |
| Elective CS |  | 0.152 |  | 0.923 |
| Sex, boy |  | 0.907 |  | 0.102 |
| Low birth weight (< 2500 g) |  | 0.061 |  | 0.0001 |
| Congenital malformation |  | 0.840 |  | 0.369 |
| Neonatal asphyxia |  | 0.024 |  | 0.141 |
| Congenital infection |  | 0.410 |  | 0.411 |

*P* values for interaction were derived from modified Poisson regression models including an interaction term between previous caesarean delivery and maternal age or history of miscarriage.

**Supplementary Table S4. Stratified analyses by maternal age.**

| Outcome |  | <35 years aRR (95% CI) | ≥35 years aRR (95% CI) |
| --- | --- | --- | --- |
| GDM |  | 1.39 (1.26–1.53) | 1.22 (1.11–1.33) |
| Preterm birth |  | 1.32 (1.14–1.53) | 1.73 (1.51–1.97) |
| Emergency CS |  | 1.09 (0.97–1.23) | 1.46 (1.30–1.65) |
| Neonatal asphyxia |  | 1.12 (0.85–1.48) | 0.55 (0.40–0.77) |

aRR, adjusted risk ratio; CI, confidence interval; CS, caesarean section; GDM, gestational diabetes mellitus.

aRRs were estimated using multivariable modified Poisson regression with robust error variance within each stratum, adjusted for BMI, education, anemia, hypothyroidism, insurance, marital status, residence, mode of conception, miscarriage history, and ethnicity (except for the stratifying variable itself).

**Supplementary Table S5. Stratified analyses by history of miscarriage.**

| Outcome |  | No miscarriage aRR (95% CI) | Miscarriage aRR (95% CI) |
| --- | --- | --- | --- |
| Placental abruption |  | 0.70 (0.49–0.98) | 1.13 (0.92–1.39) |
| Preeclampsia |  | 1.27 (0.90–1.79) | 2.41 (1.91–3.04) |
| Preterm birth |  | 1.26 (1.07–1.49) | 1.76 (1.56–2.00) |
| Emergency CS |  | 0.97 (0.85–1.12) | 1.49 (1.34–1.66) |
| Low birth weight |  | 1.04 (0.84–1.28) | 1.70 (1.46–1.99) |

aRR, adjusted risk ratio; CI, confidence interval; CS, caesarean section.

aRRs were estimated using multivariable modified Poisson regression with robust error variance within each stratum, adjusted for BMI, education, anemia, hypothyroidism, insurance, marital status, residence, mode of conception, maternal age, and ethnicity (except for the stratifying variable itself).
